# Supplementary material for: Optically Tunable Threshold Switching and Thermally Activated Transport in Planar Ag/MAPbI3 Thin Single‐Crystal Devices
Source: Adv Sci (Weinh). 2026 Aug 7;13(60):e77024. doi: 10.1002/advs.77024 (PMC13449539; doi:10.1002/advs.77024)
Supplement: Supplementary file 1 [file ADVS-13-e77024-s001.docx]

**Supporting Information:**

**Optically Tunable Threshold Switching and Thermally Activated Transport in Planar Ag/MAPbI_3_ Thin Single-Crystal Devices**

Ofelia Durante^1, §^, Valeria Demontis ^2,§,*^, Sebastiano De Stefano^1^, Selene Matta^2^, Adolfo Mazzotti^1^, Daniela Marongiu^2^, Emanuele Meloni^2^, Elisa Pili^2^, Fang Liu^2^, Nicola Sestu^2^, Angelica Simbula^2^, Mauro Carta^3^, Michele Saba^2^, Andrea Mura^2^, Massimiliano Di Ventra^4^, Giovanni Bongiovanni^2^, and Antonio Di Bartolomeo^1,*^

*^1^ Department of Physics ‘E.R. Caianiello’, University of Salerno, Via Giovanni Paolo II 132, Fisciano (SA) 84084, Italy
^2^ Department of Physics, University of Cagliari, Monserrato (CA) 09042, Italy*

*^3^ Department of Mechanical, Chemical and Materials Engineering, University of Cagliari, Cagliari, 09123, Italy
^4^Department of Physics,* [*University of California San Diego*](https://ror.org/0168r3w48)*, La Jolla, CA 92093, USA*

§shared authorship

*corresponding author: [vdemontis@dsf.unica.it](mailto:vdemontis@dsf.unica.it); [adibartolomeo@unisa.it](mailto:adibartolomeo@unisa.it)

**Index**

[**S.1** Contact polarity inversion	**2**](#_Toc197094326)

**S.2** Role of metal contact	[**3**](#_Toc197094327)

**S.3** Back-to-back Schottky diode model for dark I-V	[**4**](#_Toc197094328)

**S.4** Energy Dispersive X-ray Spectroscopy (EDS) analysis	**5**

**S.5** Richardson-type law for negative-bias branch	**6**

**S.6** Fitting parameters of the back-to-back Schottky model…........................................................**7**

[References](#_Toc197094329)	**7**

**S.1 Contact polarity inversion**

To verify the polarity-dependent nature of the effect and exclude trivial instrumentation artefacts, we repeated the I–V measurement under the same LED illumination conditions after reversing the probe connections (1→2 vs 2→1). The I–V curve is mirror-inverted (**Figure S1**), indicating that the observed asymmetry is linked to the intrinsic device polarity, i.e., to the two Ag/ MAPbI_3_ interfaces and their non-equivalent effective barriers under bias, rather than to the external measurement setup. Moreover, measurements were acquired consecutively under the same illumination settings.

**Figure S1:** I–V characteristics of an Ag/MAPbI_3_ device measured under identical LED illumination conditions at T=300 K and under pressure of 1.8 mbar with two opposite probe configurations: contact 1→2 (black) and contact 2→1 (red).

**S.2 Role of metal contact**

To evaluate the role of the contact material on the transport response of MAPbI_3_ single-crystal planar devices, we fabricated control samples using (i) Au metal electrodes and (ii) graphite-based electrodes, and compared their current-voltage characteristics in the dark and under LED illumination. Specifically, the device with gold contacts were fabricated by transferring MAPbI_3_ single crystals on prepatterned Cr/Au (5/60 nm) interdigitated metal electrodes (fabricated on Si^++^/SiO_2_ (280 µm/300 nm) substrates) using a Nitto ELP BT-150E-KL tape. Instead, the devices with graphite contacts were fabricated by directly depositing a custom made graphite conductive paste, realized by mixing 1.2 g of commercial graphite powder with 0,25 g of PMMA (polymethilmethacrylate), in 2,0 mL of chlorobenzene and leaving under stirring for 24 h.

**Figure S2:** Semi-logarithmic I–V characteristics of planar MaPbI_3_ single-crystal devices measured in the dark (black) and under LED illumination of 1.4 W (orange) for (a) Cr/Au contacts and (b) graphite contacts.

**Figure S2** shows representative semilogarithmic I-V curves acquired in the dark and under LED illumination for devices with Au (a) and graphite (b) contacts. In both cases, illumination increases the current by about 2-3 orders of magnitude in the explored bias range, indicating photo-enhanced conduction. Forward and reverse scans show no separation from the pronounced hysteretic switching activated by light observed in devices with Ag contacts. In particular, the I-V curves remain uniform and show no abrupt threshold-like transition under negative polarization in these control contacts.

**S.3 Back-to-back Schottky diode model for dark I-V**

**Figure S3** reports in linear (a) and semi-log scale (b), the experimental data and fit curve (back-to-back Schottky diode model ^[1]^) for the dark IV device characteristic at room temperature.

**Figure S3:** Dark I–V curve (orange) compared with the fit (red) for forward and (b) reverse branch obtained using a back-to-back Schottky diode model including series resistance $R_{s}$.

**S.4 Energy Dispersive X-ray Spectroscopy (EDS) analysis**

Figure S4 a) reports the scanning electron micrograph of a pristine Ag/MAPbI_3_ planar interface and b) the Ag line content profile in the direction perpendicular to the edge of the Ag electrode. In the pristine interface, the Ag signal is confined to the metal electrode region and rapidly drops to the background level in the perovskite region. Figure S4 c) and d) report the scanning electron micrograph of the Ag/MAPbI3 planar interface after electrical stress and transition to low resistance state and the Ag line content profile. In this case, the Ag signal remains slightly detectectable also in the perovskite region adjacent to the contact.

Figure S4: a) Scanning electron micrograph of a pristine Ag/MAPbI_3_ planar interface and b) Ag line content profile in the direction perpendicular to the edge of the Ag electrode. c) Scanning electron micrograph of the Ag/MAPbI_3_ planar interface after electrical stress and d) Ag line content profile in the direction perpendicular to the edge of the Ag electrode.

**S.5 Richardson-type law for negative-bias branch**

To quantify the temperature dependence, the current was also extracted from negative-bias branch at $V = - 0.5, - 1$ and $- 2 V$ and analysed within a contact-limited thermionic-emission framework following a Richardson-type law ^[2,3]^ $.$ **Figure S5** reports $ln(I/T^{2})$ versus $1/T$ where the experimental data are well described by this linearization, allowing us to extract an effective Schottky barrier height $\Phi_{B}$. From the slopes, we obtain $\Phi_{B}=0.6 \pm0.03 eV$ at $- 0.5 V$, $0.50 \pm0.02 eV$ at $- 1 V$, and $0.44 \pm0.02 eV$ at $- 2 V$ confirming the same overall picture of contact-limited thermally activated transport, extracted from positive bias, and a progressive reduction of the effective barrier with increasing bias magnitude.

**Figure S5:** Richardson plot, ln(I/T^2^) vs $1/T$ for different voltage bias (-0.5, -1, -2 V).

**S.6 Fitting parameters of the back-to-back Schottky model**

| **Fit parameter** | **Illuminated HRS** | **Illuminated LRS** |
| --- | --- | --- |
| Rs (Ω) | 1.4 $\times$10^9^ | 4 $\times$10^8^ |
| ϕB_01_ (eV) | 1.07 | 0.8 |
| ϕB_02_ (eV) | 0.8 | 0.81 |
| n1 | 1.36 | 1.04 |
| n2 | 1.04 | 1.03 |

Table S1: Main fitting parameter used to reproduce the experimental data reported in Figure 3 in the main text.

The transition from HRS to LRS is mainly captured by a decrease of the effective series resistance and by a reduction of one effective Schottky barrier, while the second barrier remains nearly unchanged. This supports a contact-mediated switching mechanism dominated by one Ag/MAPbI_3_ interface. We stress that these parameters should be regarded as effective fitting parameters describing the device state under illumination, rather than fixed microscopic constants of the two interfaces.

**References**

[1]	A. Grillo, A. Di Bartolomeo, *Adv Elect Materials* **2021**, *7*, 2000979.

[2]	A. Pelella, A. Kumar, K. Intonti, O. Durante, S. De Stefano, X. Han, Z. Li, Y. Guo, F. Giubileo, L. Camilli, M. Passacantando, A. Zak, A. Di Bartolomeo, *Small* **2024**, *20*, 2403965.

[3]	A. Sessa, T. Dadiani, S. De Stefano, O. Durante, A. Pelella, C. Parvulescu, A. Dinescu, M. Aldrigo, C. Kuo, C. S. Lue, G. D’Olimpio, E. Faella, A. Politano, M. Passacantando, A. Di Bartolomeo, *Adv Elect Materials* **2025**, *11*, e00327.
